# Imaging Single Retrovirus Entry through Alternative Receptor Isoforms and Intermediates of Virus-Endosome Fusion

**DOI:** 10.1371/journal.ppat.1001260

**Published:** 2011-01-20

**Authors:** Naveen K. Jha, Olga Latinovic, Erik Martin, Gennadiy Novitskiy, Mariana Marin, Kosuke Miyauchi, John Naughton, John A. T. Young, Gregory B. Melikyan

**Affiliations:** 1 Institute of Human Virology and Department of Microbiology and Immunology, University of Maryland School of Medicine, Baltimore, Maryland, United States of America; 2 Nomis Center for Immunobiology and Microbial Pathogenesis, The Salk Institute for Biological Studies, La Jolla, California, United States of America; Harvard Medical School, United States of America

## Abstract

A large group of viruses rely on low pH to activate their fusion proteins that merge the viral envelope with an endosomal membrane, releasing the viral nucleocapsid. A critical barrier to understanding these events has been the lack of approaches to study virus-cell membrane fusion within acidic endosomes, the natural sites of virus nucleocapsid capsid entry into the cytosol. Here we have investigated these events using the highly tractable subgroup A avian sarcoma and leukosis virus envelope glycoprotein (EnvA)-TVA receptor system. Through labeling EnvA pseudotyped viruses with a pH-sensitive fluorescent marker, we imaged their entry into mildly acidic compartments. We found that cells expressing the transmembrane receptor (TVA950) internalized the virus much faster than those expressing the GPI-anchored receptor isoform (TVA800). Surprisingly, TVA800 did not accelerate virus uptake compared to cells lacking the receptor. Subsequent steps of virus entry were visualized by incorporating a small viral content marker that was released into the cytosol as a result of fusion. EnvA-dependent fusion with TVA800-expressing cells occurred shortly after endocytosis and delivery into acidic endosomes, whereas fusion of viruses internalized through TVA950 was delayed. In the latter case, a relatively stable hemifusion-like intermediate preceded the fusion pore opening. The apparent size and stability of nascent fusion pores depended on the TVA isoforms and their expression levels, with TVA950 supporting more robust pores and a higher efficiency of infection compared to TVA800. These results demonstrate that surface receptor density and the intracellular trafficking pathway used are important determinants of efficient EnvA-mediated membrane fusion, and suggest that early fusion intermediates play a critical role in establishing low pH-dependent virus entry from within acidic endosomes.

## Author Summary

Enveloped viruses are taken up into host cells after they bind to a specific receptor on the cell surface. These viruses are often delivered to specific compartments known as endosomes which have an acid environment that can trigger the virus and endosome membranes to fuse, allowing virus entry into the cell interior. In spite of extensive studies, key steps of this process are poorly understood. To examine the mechanism of virus-endosome fusion, we took advantage of a model retrovirus (ASLV). We imaged the entry of “labeled” ASLV into cells using fluorescence microscopy and found important differences between ASLV entry, depending upon whether the virus was attached to a transmembrane receptor or to a lipid-anchored receptor. A transmembrane receptor supported faster virus uptake followed by the formation of larger and more stable fusion pores, which was somewhat delayed relative to ASLV entry into acidic endosomes. This could be due, in part, to a relatively long-lived hemifusion intermediate that preceded the formation of a fusion pore in cells expressing a transmembrane, but not a lipid-anchored receptor. These findings shed light on functional consequences of virus entry *via* distinct endocytic routes and on critical fusion intermediates leading to infection.

## Introduction

Viral fusion proteins mediate the release of nucleocapsid into the cytoplasm by merging the viral and cellular membranes. When activated by cellular receptors and/or by acidic pH, these proteins undergo extensive refolding and mediate membrane fusion (reviewed in [1], [2], [3]). Viruses that rely on low pH to activate their fusion proteins are normally confined to entry through endocytic pathways, whereas those that mediate fusion at neutral pH, following the interactions with cellular receptors, can fuse directly to the plasma membrane or to endocytic compartments [4], [5], [6], [7], [8], [9], [10]. Mechanistic studies of virus-endosome fusion have been hampered by inaccessibility of these cellular compartments and the lack of techniques to monitor the virus-endosome fusion in live cells. The mechanisms of low pH-induced fusion have therefore been extensively investigated using a cell-cell fusion model (see for example [11], [12], [13]).

Envelope glycoproteins of viruses entering cells through pH-dependent endocytic pathways are capable of mediating cell-cell fusion when expressed in the plasma membrane and exposed to acidic pH. However, recent studies revealed that productive virus entry occurs through interplay between the envelope glycoproteins and cellular factors, many of which are endosome-resident [14], [15], [16], [17], [18], [19]. The efficacy and the functional outcomes of membrane fusion in the context of cell-cell and virus-cell models can differ [8], [20], [21], [22]. For instance, endosome-resident factors, such as proteolytic enzymes [21] or lipids [20], can be essential for initiation and/or completion of the fusion process. Thus, fusion should be investigated at the physiological virus entry site(s).

Advances in live cell imaging allow the visualization of single virus entry and fusion in real time [8], [23], [24], [25]. Viral fusion has usually been visualized by incorporating relatively high amounts of fluorescent markers into their membranes, which resulted in fluorescence self-quenching [20], [23], [24], [26]. Dilution of these dyes upon virus-endosome fusion is associated with considerable increase in their fluorescence intensity, which can be readily detected. This approach detects fusion based on the lipid mixing activity, but not the actual release of the viral core/content, which is a prerequisite for infection. An alternative approach involves co-labeling the viral core and the viral membrane with different fluorescent proteins. If nearly all viral particles contain both markers, fusion can be detected based on the appearance of single-labeled puncta positive for the core marker [27], [28]. This approach allowed enumeration of the fusion events in cells pre-incubated with viruses, but not in real-time imaging experiments, perhaps due to the slow separation of the viral core and envelope. On the other hand, co-labeling the inner leaflet of viral membrane with fluorescence proteins and the membrane itself with either a lipophilic dye [8] or with tagged fusion protein [4] permitted monitoring the dynamics of viral fusion in live cells.

We have previously developed a single virus imaging-based assay for sensitive detection of early stages of fusion, from lipid mixing to formation and dilation of fusion pores [8], [29], [30]. In addition to a membrane dye, viruses were labeled with a content marker that remained trapped within virions following the proteolytic cleavage/maturation event [8], [30]. This labeling strategy provided new insights into the cellular entry site of HIV-1 [8] and helped identify key intermediates of Avian Sarcoma and Leukosis Virus (ASLV) fusion [29]. ASLV fusion proceeds in two spatially and temporally distinct steps: priming by a cognate receptor at neutral pH on the cell surface and low pH-mediated fusion with endosomes [29], [31], [32], [33], [34]. Like other acid-triggered viruses with relatively high pH threshold for fusion, such as vesicular stomatitis virus and Semliki Forest Virus [35], [36], ASLV likely fuses with early endosomes [32], [37]. However, unlike many low pH-dependent viruses, the ability of ASLV to undergo low pH-mediated fusion strictly depends on the prior priming by cognate receptors [33]. This two-step fusion mechanism makes the ASLV amenable for mechanistic studies of fusion with intracellular compartments.

Another important feature of ASLV that facilitates mechanistic studies of low pH-dependent viral entry mechanisms is that different pathways are used by subtype A ASLV depending upon usage of two natural isoforms of its TVA receptor [38], [39], [40]. The two TVA isoforms are generated from alternatively spliced mRNA: the transmembrane (TVA950) and the GPI-anchored (TVA800) receptor. Both TVA isoforms support virus infection [38], [39], but their roles in virus trafficking and fusion are not fully understood. ASLV appears to enter cells engineered to express either TVA800 or TVA950 through distinct endocytic pathways [33], [41]. This notion is supported by the following observations: (1) TVA800, but not TVA950, partitions into lipid rafts; (2) viruses entering cells via TVA800 are much more stable in NH_4_Cl-treated cells than those entering via TVA950, an effect that is compromised by lipid raft disruption; (3) cells expressing the transmembrane receptor internalize the virus more quickly but sort it into fusion-supporting acidic compartments more slowly than cells expressing TVA800.

We have previously immobilized ASLV Env-pseudotyped particles on a coverslip, overlaid permissive cells on top, and forced the virus fusion with the plasma membrane by lowering the external pH [29]. Although somewhat artificial, this configuration facilitated the visualization of single virus fusion at the cell surface. Here, we visualized ASLV Env-mediated fusion at its natural entry site. Co-labeling of the viral membrane and interior with fluorescent markers permitted real-time detection of lipid and content transfer into an endosomal membrane and into the cytosol, respectively. TVA800 less efficiently supported ASLV fusion and infection than the transmembrane TVA950 receptor. The rate of virus endocytosis, and therefore the rate of fusion, was slower and the diameter of fusion pores formed in endosomes was smaller in cells expressing the GPI-anchored receptor. We found that the level of virus infectivity in cells expressing alternative receptor isoforms correlated with its ability to form robust fusion pores. Importantly, virus fusion with endosomes progressed through a relatively long-lived hemifusion intermediate, and pores formed by this virus often remained small for several minutes. The slow progression through these steps could be rate-limiting, as evidenced by the delayed viral content release following its entry into acidic endosomes of TVA950-expressing cells. Our results thus reveal important functional implications of ASLV entry through alternative receptors and suggest that early fusion intermediates play a critical role in establishing productive infection.

## Results

### Production and characterization of pH-sensing fluorescent ASLV pseudoviruses

**Figure 1:**
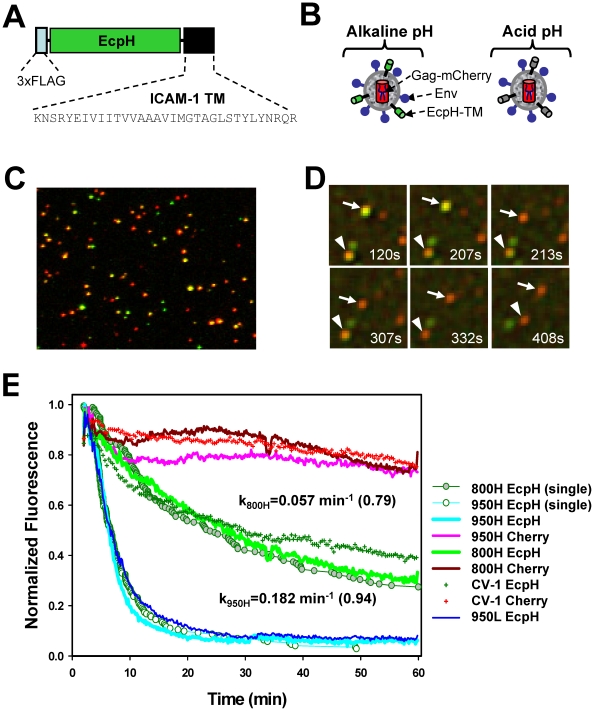
Labeling of EnvA-pseudotyped virus with a fluorescent pH-sensor and monitoring virus entry into acidic endosomes. (A) A diagram of the EcpH-TM construct that consists of the Ecliptic pHluorin (EcpH) flanked by the FLAG-tag and the transmembrane (TM) domain of ICAM-1. (B) Illustration of EnvA-pseudotyped viruses co-labeled with EcpH-TM (green) and HIV-1 Gag-mCherry (red) markers at alkaline (left) and acidic (right) pH. (C) Image of pseudoviruses co-labeled with EcpH-TM and HIV-1 Gag-mCherry. Viruses pseudotyped with EnvA were immobilized on a poly-lysine-coated coverslip and visualized as described in the Materials and Methods. (D) Images of single EnvA-pseudotyped virus entry into acidic endosomes. Disappearance of the EcpH-TM signal (green) from two individual particles (arrow and arrowhead) marks the virus delivery into acidic compartments. The Gag-mCherry-labeled viral core (red) is not considerably quenched by low pH. Time intervals (in seconds) from shifting cells to 37°C are shown. See also Video S1. (E) Integrated EcpH and mCherry signals from multiple cell-associated virions as a function of incubation time at 37°C with cells expressing TVA800, TVA950 and with parental receptor-deficient CV-1 cells. Cells were grown on coverslips, allowed to bind double-labeled pseudoviruses in the cold and washed to remove unbound viruses. Virus uptake was synchronously triggered by transferring cells into an imaging chamber pre-equilibrated at 37°C and visualized for 1 hr. Image fields encompassing multiple cells with several hundred viruses were analyzed by identifying double-labeled puncta and calculating the changes in the sums of green and red fluorescence from these puncta over time. The plots were obtained by averaging the data from 6–9 independent imaging experiments with 800H, 950L (blue line) and parental CV-1 cells and from 2 experiments with 950H cells. Error bars are not shown for visual clarity. Open and gray circles are the fractions of virions that remained unquenched at a given time point, as determined by single particle analysis for 950H and 800H cells, respectively. The waiting times from raising the temperature to the loss of EcpH fluorescence were measured for single particles, ranked and plotted as a fraction of green fluorescent virions over time. The rate constants for EcpH quenching in 800H and 950H cells determined by curve fitting the single particle quenching data are shown on the graph, along with the fractional loss of the EcpH fluorescence by the end of experiment (in parentheses). Note the slight differences in the ensemble and single particle quenching kinetics. The single EcpH quenching data were used hereafter to compare with the rates of productive endocytosis (Figure 2) and of single virus fusion (Figure 5).

To monitor EnvA-dependent endocytosis and entry into mildly acidic compartments, we labeled EnvA-pseudotyped HIV-1 virions with the pH-sensitive derivative of GFP, ecliptic pHluorin (EcpH) [42], anchored to the viral membrane *via* the transmembrane domain of ICAM-1 (Figure 1A). This construct (hereafter designated EcpH-TM) permitted sensitive detection of the pH drop below neutrality, as the fluorescence of EcpH is strongly diminished at mildly acidic pH [42] (Figure 1B). The EcpH signal from individual cell-associated viruses was lost against the background fluorescence at pH≤6.2 (data not shown). Hence, disappearance of the EcpH fluorescence in cells marks the virus entry into compartments with luminal pH around 6.2 or lower, which likely correspond to early endosomes [43]. In addition to the EcpH-TM marker, the viral core was co-labeled by appending mCherry to the C-terminus of HIV-1 Gag (referred to as Gag-mCherry).

**Figure 2:**
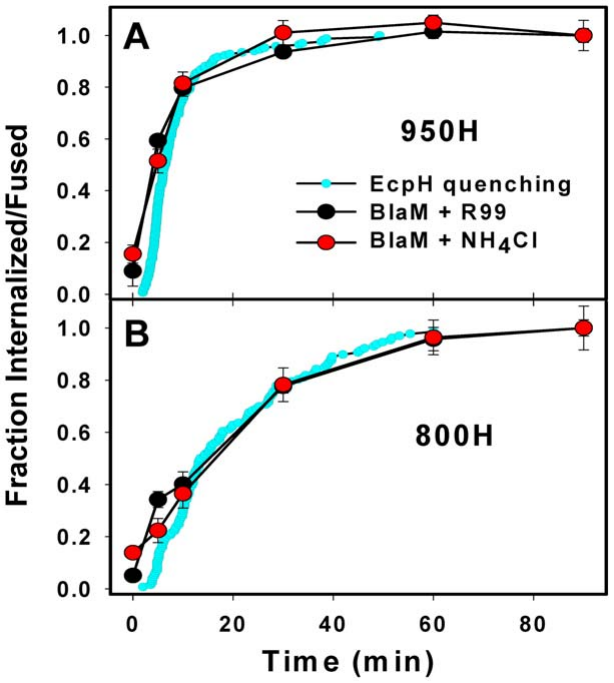
The time courses of productive EnvA-pseudotyped virus entry into cells expressing alternative receptors. (A, B) The kinetics of EcpH-TM quenching (cyan circles), productive virus uptake (black circles) and progression through low pH-dependent steps of fusion (red circles) for 950H and 800H cells, respectively. Data are normalized to the fusion signal or the extent of EcpH quenching at the last time point. Viruses were pre-bound to cells in the cold, and fusion was initiated by quickly raising the temperature. The EcpH quenching data were re-plotted from Figure 1E as a fraction of quenched virions at a given time point. Virus escape from fusion inhibitors, R99 (50 µg/ml, black circles) or NH_4_Cl (70 mM, red circles), was measured by adding the inhibitors at indicated times after shifting to 37°C. After the last time point (90 min), cells were chilled to 12°C to stop fusion, and the resulting fluorescence signal was measured after the overnight incubation at this temperature, using the BlaM assay. Data points are means of at least three independent measurements. Error bars are SEM. The kinetics of EcpH quenching and of the virus escape from inhibitors were identical in 800H cells (P>0.997), but appeared different in 950H cells (P<0.001). This difference is likely due to a 1–2 min delay in the image acquisition after moving cells into a pre-warmed buffer due to the need to find a proper image field and test the autofocus function. This delay was more evident for the faster internalizing 950H cells (panel A).

The mCherry construct was chosen because of the relative pH-independence of its fluorescence [44], which enables continuous tracking of virions entering acidic compartments. In addition, Gag-mCherry was poorly cleaved by protease upon viral maturation (Figure S1G), and, in its unprocessed oligomeric form, was not released from virions permeabilized with saponin (data not shown). Although a relatively small fraction of EnvA-pseudotyped virions underwent fusion in permissive cells (see below), the lack of Gag-mCherry cleavage further diminished the loss of red puncta and ensured a more steady reference signal for monitoring the EcpH quenching. Co-expression of Gag-mCherry and EnvA with the HIV-1 packaging vector encoding for wild-type Gag produced pseudoviruses capable of fusion (Figure S1F) and single-round infection (data not shown). Typically, ∼50% of Gag-labeled pseudoviruses were positive for the ICAM-1-based membrane marker (Figure 1C).

To determine whether EnvA is incorporated into double-labeled particles, we adhered viruses on a coverslip and stained with an EnvA-specific monoclonal antibody (Figure S1B). More than 80% of Gag-labeled particles appeared positive for Env, while only ∼30% appeared to contain all three markers. This seemingly low colocalization of Gag, Env and the membrane marker was due to a limited sensitivity of fluorescence microscopy, which did not detect a small number of ICAM-anchored fluorescent proteins on individual virions. In fact, virtually all functional virions contained EcpH-TM, since more than 96% of infectivity could be precipitated from supernatant, using anti-FLAG antibodies (Figure S1E). EcpH-TM did not appear to compete with Env incorporation (Figure S1C, D) or interfere with its function, as evidenced by similar infectious titers (usually, 10^6^–10^7^ IU/ml, Figure S1E) and the fusion activities (Figure S1F) of Gag-mCherry-labeled and double-labeled virions. Together, these results indicate that EcpH-TM did not exert detectable adverse effect on the functionality of the EnvA-pseudotyped viruses.

### EnvA pseudotyped virus is more quickly internalized when associated with TVA950

In order to elucidate the EnvA-dependent entry routes and evaluate the functional consequences of utilization of alternative receptors, we generated CV-1 cell lines stably expressing relatively high levels of TVA800 or TVA950 (designated 800H and 950H cells, respectively). CV-1 cells were chosen for their flat morphology which greatly simplified time-resolved virus imaging (see Materials and Methods). Since, the GPI-anchored isoform is always more abundantly expressed on the surfaces of CV-1 cells than the transmembrane receptor (Figure S2), it was not possible to obtain cell lines with matched surface levels of both receptors. However, as we will demonstrate below, the higher level of TVA800 expression does not affect the key findings of this paper. To examine the rate of virus uptake by cells expressing alternative receptors, we imaged entry of EcpH-TM/Gag-mCherry labeled viruses into mildly acidic endosomes based on a sudden drop of the EcpH fluorescence. As expected, the mCherry fluorescence provided a relatively stable reference signal for particles tracking (Figure 1D, E and Video S1).

Virus uptake and delivery into acidic compartments as a function of time was evaluated by analyzing the decay of the total EcpH signal from several hundred double-labeled particles in the image field. The virus uptake by 950H cells was clearly much faster than by 800H cells (Figure 1E, P<0.001). Within 15 min at 37°C, nearly 90% of virions entered acidic compartments of 950H cells, whereas only ∼30% of the EcpH signal was quenched in cells expressing the GPI-anchored receptor. The latter cells did not complete virus endocytosis even after 60 min of incubation. Surprisingly, parental CV-1 cells lacking receptors internalized the virus nearly as quickly as 800H cells (Figure 1E, green crosses and green line, respectively). Thus, in spite of the more abundant expression of TVA800 compared to TVA950 (Figure S2), the lipid-anchored receptor did not accelerate EnvA pseudotyped virus endocytosis beyond the rate of receptor-independent uptake. The modest decrease in the mCherry fluorescence over time (Figure 1E) was not due to the sample photobleaching (data not shown), but was likely caused by the limited quenching in acidic endosomes.

The EcpH quenching kinetics followed a single exponential time course and were only marginally affected by the expression level of either receptor (Figure 1E, blue vs. cyan line, and data not shown). Thus, the nature of the TVA anchorage to a cell membrane was solely responsible for the marked difference in the rate of virus endocytosis. The rate of EnvA-mediated entry into acidic endosomes was also verified by examining individual viral particles. The time intervals between placing the cells into a pre-warmed buffer and individual EcpH quenching events (e.g., Figure 1D) were measured and plotted as cumulative distributions (Figure 1E, circles). Single virus entry into acidic compartments followed the same time course as the ensemble EcpH quenching of multiple viruses in the image field. To conclude, the transmembrane-anchored TVA allowed for fast virus uptake and delivery into acidic compartments, while the GPI-anchored receptor did not accelerate virus entry into acidic endosomes compared to that in receptor-deficient, parental CV-1 cells.

### Productive EnvA-dependent virus entry is faster *via* TVA950

The EcpH quenching assay reports the EnvA-mediated virus delivery into acidic compartments, but does not directly measure the step of virus engulfment by an endosome. Also, EcpH quenching measures the bulk uptake, irrespective of whether or not it leads to EnvA-driven fusion with an endosome. To assess the rates of productive virus internalization and activation at low pH, we employed a virus-cell fusion assay, which measures the transfer of the virus-incorporated β-lactamase (BlaM) into the cytosol [45]. First, the kinetics of productive endocytosis was determined from the time course of the virus escape from the inhibitory peptide, R99. This peptide derived from the C-terminal heptad repeat region of the EnvA ectodomain is thought to block fusion by binding to a stable intermediate conformation of Env and preventing its folding into a final 6-helix bundle structure [46], [47]. Since the virus is incapable of fusing with the plasma membrane at neutral pH [29], [33], protection from R99 must occur due to its entry into endosomal compartments where it is no longer accessible to the peptide. By contrast, surface-accessible viruses that engaged receptors and thus have R99 binding sites on Env exposed would bind the peptide and lose the ability to fuse with endosome following internalization.

We pre-bound the viruses to cells in the cold and initiated their uptake and fusion by quickly raising the temperature. A fully-inhibitory concentration of R99 was added at indicated incubation times to block fusion of viruses remaining on the cell surface. Similar to the EcpH quenching kinetics (Figure 1E), viruses entering *via* TVA950 were protected from R99 faster than those utilizing the GPI-anchored receptor (Figure 2A, B). Next, EnvA activation in acidic compartments was examined through raising endosomal pH (adding NH_4_Cl) at varied times of virus-cell incubation. The extent of fusion following the addition of NH_4_Cl is a measure of progression beyond the low pH-dependent step(s) of virus entry. Irrespective of the receptor isoform, the virus acquired resistance to NH_4_Cl shortly after virus uptake, as evidenced by the identical kinetics of virus escape from R99 and NH_4_Cl (Figure 2). In other words, the time required for the internalized viruses to enter acidic endosomes and to undergo low pH-dependent conformational changes leading to fusion was too short to be resolved by this functional assay.

For a given cell line, the fraction of EnvA pseudotyped viruses that lost their EcpH fluorescence was close to the fraction of virus protected from fusion inhibitors (Figure 2). Thus, EcpH quenching provides a reasonable estimate for the productive uptake and activation of these pseudoviruses. The above results imply that, once engulfed by an endosome, the virus quickly enters acidic compartments and undergoes low pH-dependent fusion activation in both 950H and 800H cells. However, both bulk and productive virus endocytosis is markedly faster in cells expressing the transmembrane receptor compared to the GPI-anchored isoform.

### Endosomal pH can drop rapidly without significant prior virus movement

**Figure 3:**
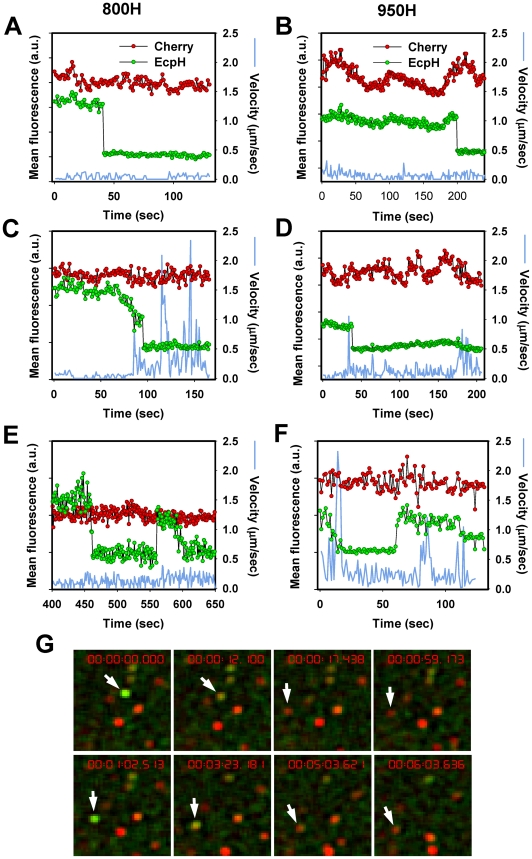
EcpH quenching patterns for individual EnvA-pseudotyped virus particles. Changes in fluorescence intensities of single virions entering 800H (A, C, E) and 950H (B, D, F) cells are shown. Single particles were tracked, using the relatively stable mCherry signal (red), and the mean fluorescence intensities of both EcpH (green) and mCherry were plotted. The instantaneous velocities of particles are shown by blue lines. (E, F) Examples of possible pseudovirus recycling events manifested by the consecutive quenching and dequenching of the EcpH fluorescence. The transient recovery of green signal likely occurs due to recycling to the cell surface or as a result of re-entry of into pH-neutral compartments. (G) Images of the transient EcpH dequenching event shown in panel F. To aid clarity, only the time interval encompassing the reversible dequenching event is represented in F and G, and an arbitrary chosen time point preceding the first EcpH decay was set to zero. See also the corresponding Video S2.

We tracked individual EcpH-TM-labeled virions to determine whether virus trafficking was a prerequisite for entry into acidic compartments. Judging by the quick EcpH transition from a fluorescent to a non-fluorescent state (Figure 3A, B, D), the majority of internalized viruses experienced an abrupt pH drop. The EcpH fluorescence of 9 out of 14 particles entering *via* TVA800 and 10 out of 11 particles entering *via* TVA950 disappeared faster than we could resolve (in less than 2 sec). The sudden loss of the EcpH signal suggests that the pH drop may occur through fusion of neutral virus-carrying endosomes with acidic compartments, as opposed to a gradual maturation of former endosomes. The quick pH drop around internalized influenza virions has been reported previously [48]. Non-instantaneous EcpH fading events appeared more common in TVA800-expressing than in TVA950-expressing cells (for example, Figure 3C). Considering that EcpH quenching occurs at pH∼6.2, which can also activate EnvA for fusion [32], [37], the vanished green fluorescence could serve as a convenient marker for the initiation of the low-pH dependent steps of fusion.

Analysis of the particle trajectories in both cell lines before EcpH quenching revealed that a large fraction of virions (>60%) entered mildly acidic compartments without undergoing quick movement or even shifting considerably from their original positions (Figure 3A, B). The lack of large-scale displacement prior to EcpH quenching and the relatively high pH-threshold of EnvA activation imply that these fusion events occur in early endosomes. We found that mobile virions moved at an average speed of 0.4±0.1 µm/sec within a 15 sec interval before or after the EcpH quenching, while their instantaneous velocity at isolated time points occasionally exceeded 1 µm/sec (Figure 3C). The apparent differences in the EcpH quenching profiles and in the virus trafficking patterns in cells expressing TVA800 and TVA950 are consistent with different virus entry pathways in these cells, however, further experiments are needed to determine whether these differences are significant.

Interestingly, the quenched EcpH fluorescence of a small fraction (<1%) of particles recovered after varied times, consistent with their reentry into neutral compartments (Figure 3E–G and Video S2). The EcpH dequenching can occur due to the virus recycling back to the cell surface, as have been proposed previously [49], while the final disappearance of green signal is likely caused by virus re-internalization. Alternatively, consistent with the partial recovery of the initial EcpH signal commonly observed for these events (Figure 3E), EnvA-pseudotyped viruses could remain internalized but enter nearly neutral intracellular compartments, perhaps recycling endosomes. Regardless of the mechanism of EcpH dequenching, these were rare events that should not contribute significantly to the dynamics of the pseudotyped virus uptake. EcpH fluorescence dequenching was transient, as the green signal vanished once again, while the mCherry fluorescence remained steady. Our data demonstrate the utility of EcpH labeling for studies of viral uptake and recycling in cells.

### Detection of single EnvA-pseudotyped virus fusion with endosomes

**Figure 4:**
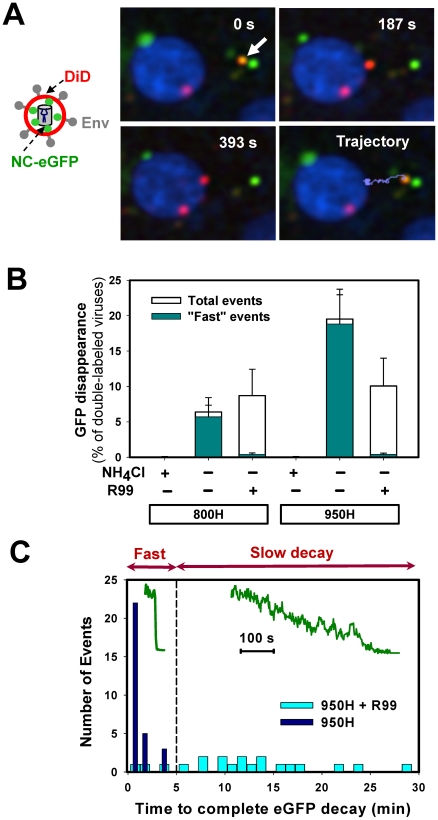
Detection of fusion between single EnvA-pseudotyped viruses and endosomal membranes. (A) EnvA-bearing pseudoviruses co-labeled with DiD (red) and NC-eGFP (green) were pre-bound to 950H cells in the cold and allowed to enter and fuse by shifting to 37°C. A double-labeled virus (arrow) underwent quick retrograde movement (light blue trajectory) and released its NC-eGFP marker near the cell nucleus, which was labeled with Hoechst-33342 (blue). See also the corresponding Video S3. (B) The loss of NC-eGFP signal from individual virions was blocked in the presence of 70 mM of NH_4_Cl, but not in the presence of 0.1 mg/ml of R99 peptide in both 800H and 950H cells (open bars). The NC-eGFP decay events that lasted less than 5 min (“fast” events) were virtually abrogated by R99 (dark cyan bars), whereas the “slow” events were still observed. The bars represent the NC-eGFP disappearance events per experiment normalized to the total number of double-labeled particles bound to cells. Error bars are SEM from at least three or more independent experiments. (C) Histograms of the time required for the complete loss of the NC-eGFP fluorescence from viruses in the presence (light cyan bars) and in the absence (blue bars) of R99. *Inset*: examples of “fast” and “slow” NC-eGFP decay events without and with the peptide inhibitor, respectively.

To measure single virus particle fusion with endosomes, we employed the virus labeling strategy used previously to visualize individual HIV-1 entry events [8] and EnvA-mediated fusion with the plasma membrane at low pH [29]. Pseudoviruses bearing ASLV Env and the Murine Leukemia Virus (MLV) core labeled with Gag-eGFP were produced and their membranes were co-labeled with DiD (a lipophilic dye emitting in far red). The MLV Gag-eGFP was chosen because, unlike the HIV-1 Gag-mCherry used for virus uptake experiments above, this precursor is cleaved by the viral protease, yielding the nucleocapsid-eGFP fragment (NC-eGFP) [30]. This fluorescent fragment becomes loosely trapped within the viral particle and is readily released from virions upon permeabilization with saponin (see below and [30]). The NC-eGFP release thus marks the formation of a relatively small fusion pore between viruses and endosomes, as evidenced by disappearance of the green signal due to its dilution in the cytosol (Figure 4A and Video S3) [8]. In contrast, DiD redistribution into an endosomal membrane results in a limited dilution of this dye and thus does not change its punctuate appearance [8].

As expected, the viral eGFP signal remained stationary when imaging experiments were carried out in the presence of NH_4_Cl (Figure 4B) or BafA1 (data not shown), which are known to block ASLV fusion through neutralizing the endosomal pH [31], [33]. However, virions were still observed to lose eGFP in the presence of fully inhibitory concentrations of R99 peptide (Figure 4B, open bars). Since even lower R99 concentrations fully blocked virus-cell fusion measured by the BlaM assay (Figure S1F), it is unlikely that the loss of green fluorescence was due to incomplete inhibition of fusion by this peptide. We reasoned that these false fusion events were likely caused by the low pH-induced quenching of NC-eGFP in virions that failed to fuse with early endosomes and eventually entered more acidic compartments. Even though the eGFP fluorescence is less pH-dependent than that of EcpH (pKa 6.0 vs. 7.0, respectively [42], [44]), the signal from virions with compromised membrane integrity should be markedly reduced at pH 5.0 typical for late endosomes (e.g., [43]).

To distinguish between false positive and true fusion events, we compared the rates of eGFP decay in the presence and in the absence of R99. This analysis revealed that the apparent loss of fluorescence occurred much more slowly in the presence of the inhibitory peptide (Figure 4C). Moreover, the eGFP decay under these fusion-blocking conditions was often incomplete (data not shown). This finding supported the notion that slow fluorescence decay was caused by low pH-dependent eGFP quenching in viruses with compromised membrane integrity. By categorizing the eGFP vanishing events into “fast” and “slow”, lasting longer than 5 min (Figure 4C), we found that, normally, the overwhelming majority of these events were fast (Figure 4B, cyan bars). This phenotype was reversed in the presence of R99: nearly all detected fluorescence decay events were slow. Consistent with the virus trafficking to late endosomes in the presence of R99, the “slow” eGFP decay events tended to occur after a longer incubation time at 37°C compared to “fast” events (data not shown). We therefore limited the imaging experiments to 35–40 min in order to further reduce the occurrence of false-positive fusion events. Thus, by disregarding the slow eGFP decay events and events occurring late after the initiation of pseudotyped virus uptake, one could be reasonably confident that eGFP disappearance marks EnvA-driven virus-endosome fusion. Based on our data, the estimated likelihood of misinterpreting an eGFP quenching event as true fusion was less than 8%.

We asked if the relatively high incidence rate of eGFP quenching in the presence of a fusion inhibitor resulted from EnvA-mediated destabilization of the viral membrane, following its activation by receptors and exposure to low pH. Increases in viral membrane permeability in the course of fusion have been previously observed in [29]. To address this possibility, viruses were immobilized on a coverslip and either pre-treated with soluble TVA ectodomain (sTVA) or left untreated. Final conformational changes in viral Env were triggered by a brief exposure to a membrane-impermeant acidic MES buffer. Since the eGFP fluorescence of sTVA-treated pseudoviruses was not significantly reduced by an acidic buffer (Figure S3), we concluded that the integrity of viral membrane was not compromised by the above treatment. Note, however, that these results do not rule out the possibility that virus permeabilization requires prolonged activation of Env with receptor and/or the presence of a target membrane.

### EnvA-mediated fusion is more rapid and efficient in TVA950-expressing cells

**Figure 5 ppat-1001260-g005:**
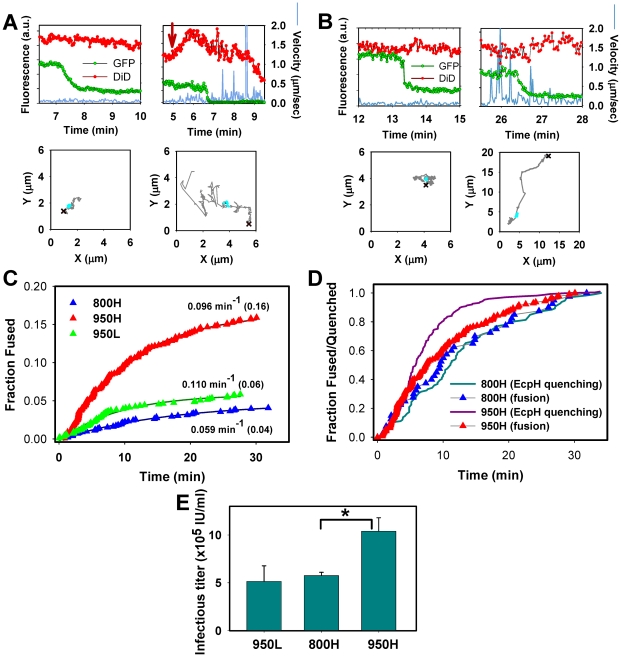
Analyses of EnvA-driven fusion with permissive cells. EnvA-pseudotyped viruses were co-labeled with NC-eGFP (green) and DiD (red), and single particle tracking was performed using the red channel. (A) Representative examples of EnvA-mediated fusion with 950H cells without (left) and with (right) significant particle motility associated with the NC-eGFP release. Red arrow marks the increase in red signal due to the DiD fluorescence dequenching. (B) Virus fusion with 800H cells without and with prior quick movement. The particles' trajectories are shown on lower panels. Red crosses mark the beginning of trajectories, while cyan circles mark the segment where the NC-eGFP signal was lost. (C) The kinetics of virus fusion with 800H cells (blue), 950H (red), and 950L (green) cells. The time intervals from shifting to 37°C and the onset of the NC-eGFP release (fusion) from individual particles were measured, ranked and plotted as cumulative probability distributions after proper normalization. The final time points were normalized according to the probability of fusion with different cells determined by single virus imaging: 0.16 for 950H, 0.04 for 800H and 0.06 for 950L cells (shown in parentheses). The resulting plots were fitted with single exponential functions (solid lines), and the obtained exponential coefficients are shown by each plot. (D) Comparison of the EcpH quenching kinetics (solid lines) and the single virus fusion kinetics (triangles). Shown are the EnvA-mediated fusion data from panel C along with the EcpH quenching data re-plotted from Figure 2 (triangles and solid lines, respectively). Symbols in panels C and D are colored identically. To aid comparison, all measurements were normalized to the respective signals at the last time point. (E) EnvA-pseudotyped virus infectivity in CV-1 cells expressing alternative receptors. Cells expressing a reduced level of TVA950 are designated 950L. In these experiments, pseudoviruses consisting of EnvA and the core of MLV encoding β-Gal were used. TVA800- or TVA950-expressing cells were incubated with the same amount of viral inoculum in the cold to allow virus binding, washed and incubated for 1.5 hr at 37°C to allow infection. Viral fusion was stopped by adding R99 peptide (50 µg/ml), and cells were maintained for 2 days prior to determining the infectious titer, using a β-Gal assay. A representative experiment with triplicate measurements is shown. Error bars are standard deviations. Asterisk indicates a significant difference in virus infectivity in 800H and 950H cells (P<0.047).

We tracked single viruses and analyzed the extent and the rate of their fusion in cells expressing alternative receptors, using the above “time of eGFP decay” criterion to discern true fusion from the eGFP quenching by low pH. Similar to the EcpH quenching dynamics (Figure 3), single viruses exhibited diverse movement patterns prior to fusion, ranging from little or no displacement to fast and often directional movement (Figure 5A, B). These movement patterns were observed irrespective of the receptor isotype, but particles trafficked by TVA800 appeared to undergo more sustained fast movement prior to fusion than those internalized *via* TVA950 (data not shown). A more detailed analysis of pseudovirus trafficking *via* alternative receptors is beyond the scope of the current paper which is instead focused on the endosomal fusion events mediated by these receptors.

The efficiency of virus fusion was determined by normalizing the number of individual fusion events in each experiment to the number of cell-bound double-labeled virions at the onset of image acquisition. This analysis showed that 16±2% (n = 9 independent experiments) of viruses fused with 950H cells, 4±1% (n = 7) with 800H cells and 6±2% (n = 4) with cells expressing a lower level of TVA950 (referred to as 950L, see Figure S2). Since only ∼65% of cell-bound viruses are internalized by 800H cells in imaging experiments lasting about 35 min (Figures 1E and 2A, B), a longer incubation time to allow complete virus uptake would increase the extent of fusion to 6%. However, even this corrected extent of fusion is significantly lower than that in 950H cells (P<0.003). To conclude, TVA950 supported more efficient EnvA-mediated fusion than did TVA800.

We then determined the kinetics of EnvA-driven fusion in cells expressing TVA950 and TVA800 by measuring the waiting times from raising the temperature to the onset of “quick” eGFP release from individual particles (Figure 5C, red vs. blue triangles). These results were normalized to reflect the extents of fusion with respective cell lines, as described in the previous paragraph (see also Figure 4B). To ease the comparison of the fusion kinetics, experimental data were fitted with a single exponential function. This analysis showed that the fusion rate was 1.6 times faster in 950H compared to 800H cells (0.096±0.001 vs. 0.059±0.004 min^−1^). Although the extent of fusion was reduced in 950L cells expressing a lower density of TVA950 compared to 950H cells, the rate of fusion was not affected (0.110 min^−1^, Figure 5C, green triangles).

The above results show that the rate of fusion with 950H cells (0.096±0.001 min^−1^) is almost 2-fold slower than its uptake and delivery into acidic compartments (0.182±0.004 min^−1^, Figures 1E and 2A). By contrast, virus uptake by and fusion with 800H cells occurred with identical kinetics (0.057±0.001 vs. 0.059±0.004 min^−1^, respectively). The above relationships are better illustrated by plotting side-by-side the EcpH quenching and fusion kinetics (Figure 5D, solid lines vs. triangles, respectively). Thus, fusion with 800H cells was not rate-limiting as compared to the slow virus endocytosis through this receptor. By contrast, fusion with 950H cells appeared to occur after a measureable delay following the quick virus entry into acidic compartments. Delayed fusion after the virus delivery into acidic compartments of 950H cells, but not 800H cells, further supports the notion that EnvA-pseudotyped particles are routed through different endocytic pathways in cells expressing these receptor isoforms.

We then asked whether the greater efficiency of EnvA-mediated fusion (content release) in 950H cells compared to 800H cells bears relevance to infection. Single-cycle infectivity measurements [50] revealed that the virus' ability to infect 950H cells was ∼2-fold higher compared to cells expressing TVA800 (Figure 5E). The lower virus titer in 800H cells was not caused by a less efficient binding to these cells compared to 950H cells, as determined by the p24 ELISA assay (data not shown). Since 950L cells expressing a reduced level of the transmembrane receptor internalized pseudotyped virus nearly as quickly as 950H cells (Figure 1E) and yet did not support the same level of infection (Figure 5E), the rate of endocytosis did not seem to determine the level of viral infectivity. Instead, infectivity appeared to correlate with the efficiency of the viral content release (Figure 5C).

### EnvA can mediate the formation of long-lived small fusion pores

**Figure 6:**
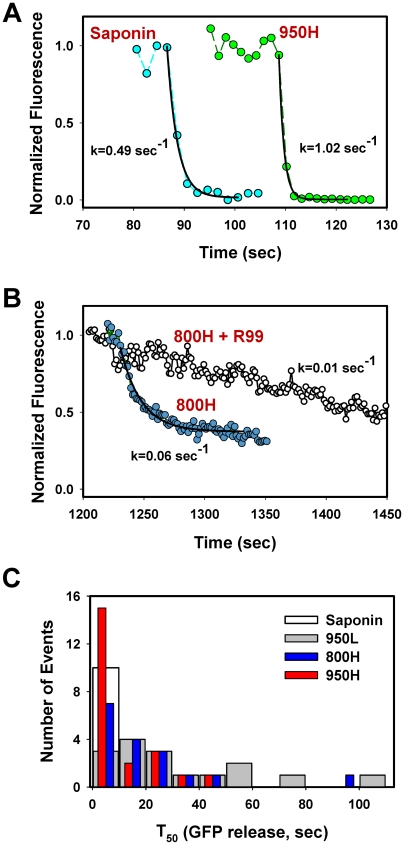
Analysis of the rate of NC-eGFP release from single viruses fusing with endosomes. (A) The NC-eGFP release from representative EnvA-pseudotyped virus particles upon treatment with 0.1 mg/ml saponin (cyan circles) and as a result of fusion with a 950H cell (green circles). Exponential fits (solid black lines) of the decaying green fluorescence and the obtained decay rates are shown. (B) Similar to panel A, but for the virus fusion with 800H cells in the absence (blue circles) or in the presence (open circles) of 0.1 mg/ml R99 peptide. (C) Half-times (T_50_) of the NC-eGFP release in 950H (red bars), 950L (gray bars) and 800H (blue bars) cells. The T_50_ values were determined from the exponential decay coefficients, as shown in panels A and B. The distribution of T_50_ for saponin-formed lytic pores in single virions is shown by an open bar.

The eGFP decay events marking the EnvA-driven virus fusion lasted anywhere from a few seconds to minutes (Figures 4–6). These highly variable rates of viral content release were not related to the differences in retention of NC-eGFP by individual viral cores. Indeed, saponin-treated viruses lost their green fluorescence within a few seconds after the onset of lysis (Figure 6A, cyan circles), consistent with the ability of saponin to form relatively large (3–10 nm) pores in membranes [51], [52]. These rather uniform rates of NC-eGFP release by saponin (Figure 6C, open bar) demonstrate that the vast differences in the release rates during fusion are due to the variable sizes of fusion pores which limit the efflux of viral content. Thus, in agreement with previously published studies [8], [30], NC-eGFP does not appear to bind to any significant extent to the viral core, so that its release time is determined by the pore diameter.

**Figure 7:**
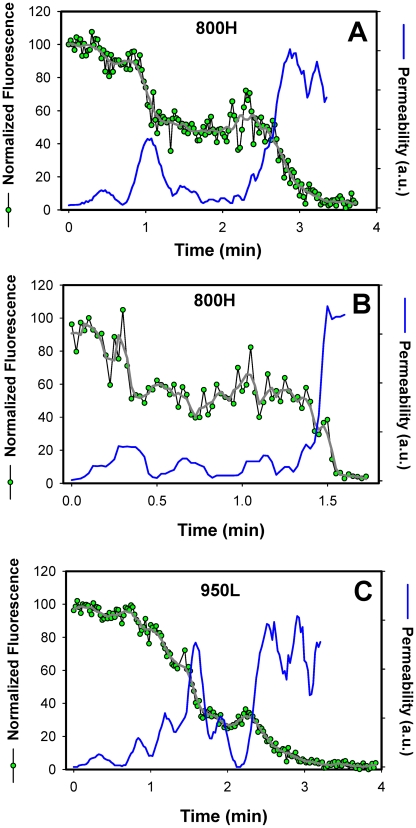
Transient openings of fusion pores formed by EnvA in endosomes. The NC-eGFP release profiles (green circles) in 800H (A, B) and 950L (C) cells was determined by single particle tracking, using the red DiD signal as reference (not shown for clarity). The decay in the total eGFP fluorescence of a single particle was normalized to the green signal prior to fusion (at arbitrary time = 0). After smoothing the traces (gray lines), the effective permeability (P) of a fusion pore was calculated, using the equation P = (dF(t)/dt)/F(t), where F(t) is the normalized eGFP fluorescence intensity of a virion as a function of time. The obtained pore permeability traces (blue lines) show reversible opening and closure of fusion pores.

The above results show that the relative size of fusion pores can be estimated based on the rate of NC-eGFP release. The majority of these events followed a single exponential time course (Figure 6A, B). A small fraction of particles lost their green fluorescence too quickly to be kinetically resolved or exhibited complex NC-eGFP release profiles (e.g., Figure 7). We therefore fitted the eGFP fluorescence decay with an exponential function and used the obtained parameters to calculate the time required to release half of the viral content (T_50_, Figure 6C). The half-times ranged from 2 sec or less for the largest fusion pores to over a minute for smaller pores that restricted the release of the fluorescent marker. An average fusion pore formed in 950H cells was considerably larger (i.e., the release half-times were shorter) than in 800H cells (P<0.043, Figure 6C). Interestingly, the rate of eGFP release diminished as a result of modest reduction in the TVA950 expression in 950L cells (P<0.001); the average pore sizes in 950L and 800H cells were not significantly different (P>0.17). As discussed above, the addition of R99 drastically increased the half-time of fluorescence decay (Figures 4C and 6B), supporting the notion that the aberrantly slow eGFP fading was caused by trafficking of the inhibited virions to acidic late endosomes and not by fusion. We thus concluded that: (1) EnvA forms smaller fusion pores in cells expressing the GPI-anchored receptor compared to those expressing the transmembrane receptor; and (2) the average pore diameter is proportional to the TVA950 expression level.

A single-exponential decay of the eGFP fluorescence suggests that the size of a fusion pore remained relatively constant throughout the release process. Had the pore shrunk or enlarged during the release, the fluorescence decay rate should have slowed down or accelerated, respectively, thereby deviating from an exponential time course (as illustrated in Figure 7). Thus, a single-exponential release lasting a few minutes implies that a nascent fusion pore was small and surprisingly stable. Note that these measurements likely underestimate the lifetime of a small pore, since the pore evolution cannot be monitored once NC-eGFP is released. It therefore appears that small fusion pore between the virus and an endosome are often long-lived. Once formed, fusion pores may remain small up for several minutes.

Analysis of the viral NC-eGFP decay profiles in different target cells revealed that not only did TVA800 support the formation of smaller pores, but that these pores were also less stable than in TVA950-expressing cells. A fraction of eGFP release events in 800H cells exhibited a biphasic behavior suggestive of transient closing and reopening of a pore (Figure 7A, B). This phenomenon is referred to as pore flicker [53]. The cessation of NC-eGFP release after the first drop in fluorescence corresponds to closure or shrinkage of pores to sizes that do not permit the passage of this marker, while resumed release marks the pore reopening. Of course the NC-eGFP efflux from virions does not differentiate between the formation of a single and multiple fusion pores. It is therefore possible that the resumed fluorescence decay is not due to re-opening of the same pore, but rather due to the formation of a new one. We consider the possibility of multiple pore formation for a single retroviral particle less likely because these viruses incorporated relatively few fusion proteins and fuse with low probability [29].

Transient fusion events were also observed in cells expressing a lower density of TVA950 (Figure 7C), albeit less frequently than in 800H cells. To better illustrate the dynamic nature of fusion pores formed by ASLV Env, we calculated their relative permeability (Figure 7, blue lines) based on the eGFP decay profile, as described in [54]. EnvA-driven fusion pores formed in 800H and, less frequently, in 950L cells clearly opened and closed transiently prior to releasing the entire viral content. Collectively, the above results imply that TVA800 not only failed to quickly deliver the virus into acidic endosomes, but also did not support efficient fusion. These findings are especially striking considering that TVA800 was over-expressed relative to TVA950.

### EnvA-driven fusion is delayed relative to lipid mixing

**Figure 8:**
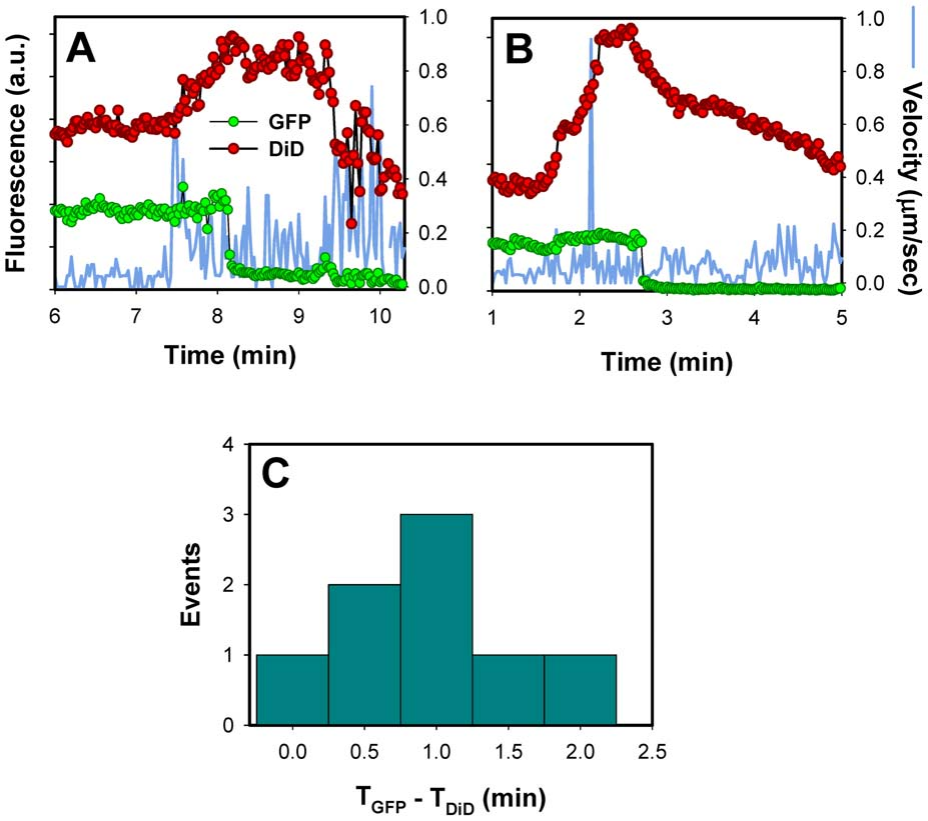
Lipid mixing step precedes the release of the viral content into the cytosol. (A, B) Single EnvA-pseudotyped particles labeled with NC-eGFP and DiD were tracked and the total fluorescence intensities of these markers were plotted (green and red circles, respectively). Blue traces are the instantaneous velocities of viral particles. DiD dequenching is manifested in the increased red fluorescence intensity. The decrease of the DiD fluorescence after dequenching is likely caused by membrane trafficking that removes the lipid dye from a recipient endosome. (C) The distribution of lag times between the increase in DiD intensity (T_DiD_) and the onset of NC-eGFP release (T_GFP_).

Virus-endosome fusion has been visualized by incorporating relatively high, self-quenching concentrations of a lipophilic dye into viruses by direct injection into viral suspension and following individual dequenching events [20], [23], [24], [26]. The virus labeling strategy employed in this work was based on labeling the virus producing cells (see Materials and Methods) with little or no control over the amount of DiD incorporated into the viral membrane. Nevertheless, we did detect a marked increase of the DiD signal for a fraction of pseudoviruses that incorporated a greater amount of this dye (Figure. 5A, red arrow, and Figure 8A, B).

**Figure 9:**
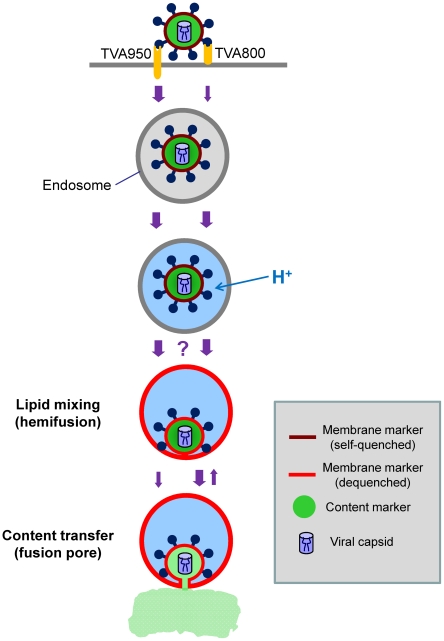
Schematic illustration of ASLV entry and fusion through alternative receptors. The viral membrane (DiD, red) marker redistributes to the limiting membrane of an acidic endosome as a result of hemifusion. Transition from dark red to light red color illustrates the dequenching of the DiD fluorescence upon its dilution within the membrane of a recipient endosome. Subsequent formation of a small fusion pore permits the release of a viral content marker (green) into the cytosol. The ASLV entry/fusion pathway *via* the transmembrane TVA950 receptor is shown by the left series of arrows, while its entry through TVA800 is depicted on the right. The relative rates of ASLV endocytosis and of intermediate steps of fusion are indicated by the width of arrows. The transient opening of small fusion pores seen in TVA800-expressing cells is schematically shown as the reversal to a hemifusion stage. The question mark reflects the lack of data of the rate of transition from low pH-activation to hemifusion, as well as the inability to detect DiD dequenching in cells expressing TVA800. The slow transition from hemifusion to a small pore in 950H cells is illustrated by a thin arrow.

DiD dequenching allowed, for the first time, the distinction between lipid and content mixing steps of virus fusion with endosomes. DiD transfer into an endosomal membrane showed that fusion had reached at least a hemifusion stage, which is operationally defined as lipid mixing without content transfer [55] (see Figure 9 below). It is possible, however, that this lipid transfer occurs through a nascent fusion pore that is too small to allow the passage of NC-eGFP; subsequent dilation of such small pore would lead to detectable content release. Analysis of DiD dequenching showed that these events always preceded the eGFP fluorescence decay and that the average lag time between lipid and content transfer was around 1 min (Figure 8C). This result demonstrates the existence of a long-lived fusion intermediate upstream of formation of detectable pores. If DiD redistribution that precedes the NC-eGFP release occurs in the absence of pore formation, our finding would support the existence of relatively stable hemifusion intermediate *en route* to fusion.

Interestingly, for the same batch of labeled virus, DiD dequenching was almost exclusively observed in cells expressing a high level of TVA950, but not in 800H or 950L cells. It is possible that the lack of DiD dequenching in 800H cells was simply due to slow lipid transfer between the virus and a recipient endosome. In this case, the increase in the overall DiD fluorescence could be prevented/masked by its concomitant removal from a recipient endosome through membrane trafficking. The pronounced decrease in the DiD signal that follows its dequenching (Figure 8) supports the notion that a recipient endosome loses a large fraction of this marker within a few minutes. It thus appears that the architecture of pre-fusion sites formed by EnvA in cells expressing alternative receptors could be different with respect to their permissiveness for redistribution of a lipophilic marker.

In summary, by monitoring the transfer of membrane and content markers during single EnvA-pseudotyped virus-endosome fusion, we were able to identify a long-lived hemifusion-like intermediate in 950H cells. Small and surprisingly stable fusion pores could represent another intermediate of ASLV fusion, however, we cannot rule out the possibility that these pores never fully dilate and thus do not lead to productive infection. The surprising longevity of these small pores in spite of high levels of receptor expression suggests that these steps could be rate-limiting for ASLV entry, in agreement with the slower rate of fusion compared to virus activation in acidic endosomes of 950H cells (Figure 5). The rather steep dependence of the average size and stability of fusion pores on the surface density of TVA950 indicates that, in natural target cells expressing lower levels of both receptors, progression through fusion intermediates could be even slower.

## Discussion

In this study we used a model retroviral Env-receptor system to dissect the intermediate steps of virus entry and fusion with endosomes. The requirement for EnvA priming by cognate receptors on the cell surface followed by low pH-dependent fusion with endosomes facilitates the identification of critical intermediates of ASLV entry. Furthermore, the ability of the subgroup A virus to utilize two natural receptor isoforms differing only in their membrane anchors permitted the comparison of distinct entry pathways. As discussed below, the results of this paper have broad implications for the mechanisms of virus entry through endocytosis. Our findings reveal that receptor density and endosomal trafficking pathways could play critical roles in the virus' ability to form functional fusion pores and, therefore, to establish productive infection.

First, the time course of receptor-mediated endocytosis was determined by adding the inhibitory peptide R99 which targets the late step of fusion. Second, delivery of internalized pseudoviruses into mildly acidic compartments was monitored, using particles tagged with the pH-sensitive GFP variant, EcpH. Third, the progression of fusion beyond the low pH-dependent steps was measured based on the virus escape from inhibition by NH_4_Cl. Fourth, single virus fusion events were visualized in real time based upon the release of a fluorescent marker trapped within the viral membrane. Fifth, the relative size and the stability of fusion pores were evaluated based upon the rate of viral content redistribution. Sixth, a long-lived hemifusion-like intermediate of single EnvA-pseudotyped virus fusion with endosomes was identified from the temporal differences in transfer of viral lipid and content markers.

We found that both bulk and productive virus endocytosis were much faster through TVA950 than through TVA800, although the latter receptor was more abundantly expressed in target CV-1 cells. Different rates of virus internalization *via* alternative receptors are in full agreement with those reported for HEK 293 cells [41] and are consistent with distinct routes of virus trafficking mediated by these receptors. In addition, we found that TVA800 did not noticeably accelerate endocytosis of EnvA-pseudotyped viruses compared to their non-specific uptake by parental cells lacking the receptor. Failure of TVA800 to facilitate the virus uptake could reflect its slow recruitment by viruses, perhaps due to restricted lateral diffusion of this receptor, as proposed in [49]. Alternatively, the rate of virus uptake could be determined by the rate of receptor endocytosis. Quick internalization *via* TVA950 could be aided by receptor-mediated signaling and recruitment of endocytic machinery.

Once internalized by CV-1 cells expressing either receptor isoform, viruses quickly entered into acidic endosomes and progressed through the low pH-activation steps (Figures 1E, 2 and 5). In HEK 293 cells, however, the slower uptake *via* TVA800 was compensated by faster escape from NH_4_Cl inhibition as compared to TVA950 cells [41], perhaps due to a faster virus delivery into acidic compartments. The quicker pace of post-internalization steps of fusion in CV-1 cells was likely caused by the high density of receptors, as these steps tended to be slower in cells expressing reduced levels of receptors (Figure S4).

The comparison of the kinetics of EcpH quenching, low pH-activation of EnvA (escape from NH_4_Cl) and individual fusion events revealed that virus internalization was rate-limiting for fusion with cells expressing the GPI-receptor. In these cells, viral uptake, low pH activation, and content release occurred at the same rate (Figures 1E and 2B vs. 5D). On the other hand, EnvA-mediated fusion with cells expressing transmembrane receptor was delayed compared to its low pH activation, suggesting the existence of slow post-internalization steps. We thus surmise that TVA800 does not accelerate endocytosis of this virus, but quickly traffics internalized particles into acidic fusion-permissive compartments. By contrast, TVA950 appears to mediate quick virus uptake and delivery into acidic compartments which allow Env activation but do not support fusion (Figure 9). Only later do low pH-activated (perhaps even hemifused) viruses enter fusion-permissive compartments in 950H cells and release their content. The delayed virus fusion could be due, in part, to a long-lived hemifusion intermediate that precedes the formation of a fusion pore in 950H, but remained undetected in 800H cells (Figure 8). Interestingly, Dengue virus fusion has been recently reported to be delayed relative to its low pH activation [56].

The potential problem with relating the bulk virus uptake (EcpH quenching) to fusion is that the majority of internalized virions fail to release their content from within endosomes. However, the closeness of the kinetics of productive virus endocytosis and of its bulk uptake (Figure 2) justifies the use of the fluorescence quenching assay. Clearly, a better approach to studying productive entry would be to monitor entry into acidic compartments and fusion of the same viral particle. The labeling and imaging techniques that could accomplish this task are currently under development.

Imaging experiments revealed a long-lived intermediate, likely a hemifusion, which preceded the viral content release by about 1 min (Figure 8). Although DiD dequenching was not detected in 800H and 950L cells, this does not rule out the existence of a hemifusion intermediate in these cells. Due to the removal of lipophilic dyes through membrane trafficking, lipid transfer to a recipient endosome must be relatively fast to result in appreciable increase in the local DiD fluorescence. We surmise that a hemifusion-like intermediate in TVA800-expressing cells may restrict lipid diffusion and therefore remain undetected. Indeed, it has been shown that hemifusion intermediates and even nascent fusion pores formed by viral proteins can restrict lipid diffusion [12], [57].

The second entry step that could represent a long-lived intermediate of EnvA-mediated fusion was the formation of a small pore. At this point, we cannot rule out the alternative possibility that long-lived small pores are simply off-path structures that do not lead to productive infection. However, by analogy with the fusion pores formed at the cell surface [29], it is plausible that at least a fraction of small pores formed in endosomes fully enlarges. To restrict efflux of the loosely trapped NC-eGFP marker, the pore diameter should be comparable to the size of this marker. The NC fragment is approximately 3-fold smaller than eGFP molecule, which has the dimensions of 4 by 3 nm [58]. Therefore, the NC should not considerably increase the overall size the NC-eGFP marker, suggesting that small fusion pores formed by EnvA should be a few nanometers in diameter. This size is consistent with the diameter of a saponin pore (3–10 nm [51], [52]) which does not considerably restrict the release of viral content (Figure 6).

Perhaps the most surprising finding of this work was the marked difference in early fusion pores formed by EnvA in cells expressing alternative receptors. Both the apparent diameter and stability of early fusion pores were greater in 950H compared to 800H cells (Figures 6 and 7). These differences could indicate the role for the TVA950 transmembrane domain in forming and/or stabilizing a fusion pore. However, TVA appears to dissociate from the EnvA at neutral pH when EnvA interacts with target membrane [59], [60] and is therefore unlikely to be a part of an endosomal fusion complex. An alternative explanation for the differences in fusion pores in cells expressing TVA isoforms is that these structures are formed in distinct endosomal compartments or in distinct lipid microdomains of the same endosomal compartment. It is conceivable that different compositions and/or dynamics of endosomal compartments harboring the virus in 800H and 950H cells can determine the properties of these pores. Yet another explanation for the unstable fusion pores formed through TVA800 is the less optimal priming of ASLV Env by this receptor. It has been shown that suboptimal conditions for fusion (e.g., pH, temperature, density of active fusion proteins) preclude the formation of pores capable of enlarging and releasing the nucleocapsid [12], [57], [61], [62]. This notion is further supported by the reduced pore diameter (Figure 6) and stability (Figure 7) in cells finding expressing a lower level of TVA950.

The delayed virus uptake and lower efficiency of fusion and infection in 800H compared to 950H cells imply that ASLV is more likely to enter natural target cells expressing both isoforms *via* the transmembrane receptor. Moreover, the usage of the GPI-anchored receptor in these target cells should be further minimized, since these cells express lower levels of this isoform compared to TVA950 [39]. The higher infectivity of EnvA-pseudoviruses in cells expressing the transmembrane receptor compared to the GPI-anchored isoform could be traced back to the formation of more robust fusion pores and of an upstream hemifusion-like intermediate that did not noticeably restrict lipid redistribution. Our data thus imply that the size and the stability of endosomal pre-fusion and early fusion sites depend on the TVA receptor isoform and that these early structures determine the outcome of ASLV entry.

## Materials and Methods

### Cell lines, viruses and reagents

CV-1 cells and HEK 293T/17 cells were obtained from ATCC (Manassas, VA) and grown in Dulbecco's modified Eagle high glucose medium (DMEM, Invitrogen Carlsbad, CA) supplemented with either Cosmic bovine serum or fetal bovine serum, respectively, purchased from the Hyclone Laboratories (Logan, UT). CV-1 cells were transduced with VSV-G pseudotyped retroviral vectors pCMMP-TVA950 or pCMMP-TVA 800 as described previously [41]. Cells expressing either high or low levels of either TVA receptor were sorted by flow cytometry using a FACS Vantage SE DiVa (BD Biosciences; Salk CCMI Core Facility) after binding to a subgroup A ASLV SU-IgG fusion protein and a FITC-conjugated secondary antibody (swine anti-rabbit FITC) [63]. Stable expression levels of the receptors were verified several times by flow cytometric analysis over the course of these studies.

The fluorescent MLV core (Gag-eGFP and Gag-Pol) was pseudotyped with the ASLV EnvA glycoprotein lacking the cytoplasmic domain (designated EnvAΔCT) and co-labeled with a membrane marker DiD, as described previously [29] with minor modifications. Briefly, HEK 293T cells on a 6 cm dish were transfected with 3 µg Gag-eGFP, 10 µg Gag-Pol, 20 µg MLV LTR-LacZ and 20 µg ASLV EnvAΔCT expressing vectors, using the Ca-phosphate protocol. Transfected cells were labeled with 5 µM DiD (freshly dispersed in pre-warmed serum-free OptiMEM) on the following day. Cells were kept in a CO_2_ incubator for 4 hr, washed and incubated for additional 24 hr in a full growth medium. Virus-containing extracellular medium was collected 48 hr post-transfection, briefly centrifuged to remove cell debris, and passed through 0.45 µm filters. Virus preparations were aliquoted and stored at −80°C. The infectious titer of EnvAΔCT-pseudotyped viruses was determined using the β-Gal assay, as described before [8].

For the virus uptake measurements, HIV-1 Gag fused at the C-terminus to the mCherry sequence (see below) was pseudotyped with EnvAΔCT and labeled with the pH-sensitive EcpH-TM construct. These pseudoviruses were produced by transfecting a 10 cm dish of HEK 293T/17 cells with plasmids encoding HIV-1 R8ΔEnv (3.3 µg, from C. Aiken, Vanderbilt University), 1 µg pcRev, HIV-1 Gag-mCherry (1 µg), EcpH-ICAM-1 (3.3 µg), and EnvAΔCT (3 µg), using PolyFect Transfection Reagent (Qiagen, Valencia, CA).

Sodium pyruvate was from Sigma (St. Louis, MO), penicillin-streptomycin from Gemeni Bio-Products (West Sacramento, CA), L-Glutamine from Lonza (Walkersville, MD), phosphate-buffered saline (PBS) and geneticin were from Mediatech Inc. (Manassas, VA). The far red lipophilic dye DiD, Hoechst-33342 nuclear stain and MitoTracker Deep Red were purchased from Invitrogen (Carlsbad, CA). The EnvA-derived R99 peptide (>95% purity by HPLC) was synthesized by Macromolecular Resources (Fort Collins, CO).

### Construction of HIV Gag- and ICAM-1-based fluorescent markers

To construct the HIV Gag-mCherry expression plasmid, the codon optimized HIV-1 Gag fragment [64] was amplified by PCR using the following primers: TAAGCTTGCC ACCATGGGCG CCCGCGCCAG CGTGCTGAGC and TGGATCCCTG GCTGCTGGGG TCGTTGCCGA ACAGGCT. Hind*III*-BamH*I* fragments of the PCR products were cloned into pcDNA3.1zeo (+) vector (Invitrogen,Carlsbad, CA). The mCherry coding sequence was amplified by PCR using the pRSET-BmCherry vector (from R. Tsien, University of California, San Francisco) as a template and primers AGGATCCAAG GGCGAGGAGG ATAACATGG and ACTCGAGTTA CTTGTACAGC TCGTCCATGC CGCCGGTGGA GTGGC. BamH*I*-Xho*I* fragments of the PCR products were cloned into the pcDNA3.1 zeo (+)-HIV gag plasmid. To construct the EcpH-TM expression plasmid, the fragments of ecliptic pHluorin (EcpH) [42] were amplified by PCR using the following primers: TAAGCTTCTC GAGAGTAAAG GAGAAGAACT TTTCACTGG and TGAATTCTTG TATAGTTCAT CCATGCCATGTG. Hind*III-*EcoR*I* fragments of the PCR products were cloned into p3xFLAG CMV9 vector (Sigma, St. Louis, MO). The transmembrane fragment of ICAM-1 was amplified by PCR using the pCDM8 ICAM-1 vector (Addgene Inc., Cambridge, MA) as a template and the following primers: AGAATTCACG GTATGAGATT GTCATCATC and TGGATCCTCA CCGCTGGCGG TTATAGAGGTA. EcoR*I*-BamH*I* fragments of the PCR products cloned into p3xFLAG CMV9-EcpH vector.

### Virus-cell fusion

EnvA-mediated virus-cell fusion was measured using the β-lactamase (BlaM) assay, as described previously [8], [65]. Briefly, pseudoviruses were prepared by transfecting HEK 293T/17 cells with 2.4 µg pR8ΔEnv, 1 µg BlaM-Vpr expressing pMM310 vector [66], 1 µg pcRev, and 3 µg of ASLV EnvAΔCT-expressing plasmid, using the PolyFect transfection reagent. Viruses were bound to target CV-1 cells by centrifugation at 2095×g, 4°C for 30 min. After washing off unbound viruses, cells were incubated at 37°C for 90 min in the presence or absence of 50 µg/ml R99 peptide. Cells were then loaded with fluorescent CCF2-AM substrate (Invitrogen) and incubated overnight at 12°C. Intracellular â-lactamase activity (ratio of blue to green fluorescence) was measured using the Synergy HT fluorescence microplate reader (Bio-Tek, Germany).

### Imaging single virus uptake and fusion

Virus uptake and delivery into mildly acidic compartments was assessed using particles co-labeled with HIV-1 Gag-mCherry and EcpH-TM as the viral core and membrane markers, respectively. EnvA-driven fusion was measured using pseudoviruses co-labeled with MLV Gag-eGFP and DiD. Cells were grown to confluency on No. 0 glass coverslips using phenol red-free growth medium. Cells were washed with Hanks' Balanced Salt Solution (HBSS) supplemented with 1 mM sodium pyruvate and 2% serum. Viruses were diluted in this buffer and spinoculated onto cells at 2100×g (4°C) for 20–30 minutes. Free viruses were removed by washing, and cells were placed on ice and used for imaging experiments within 3 hr. Pieces of a coverslip with cells were transferred into a glass-bottom imaging chamber pre-warmed to 37°C thereby initiating the virus uptake and fusion. Image acquisition started immediately after finding a suitable image field, which usually took about 30 sec after transferring cells into the chamber. Image acquisition started after a longer delay (up to 2 min) after transferring the cells in experiments where virus endocytosis was monitored by EcpH quenching.

Imaging was performed using the Personal DeltaVision imaging system (Applied Precision LLC., Issaquah, WA) equipped with an environmental enclosure that maintained the desired temperature and humidity around the sample. An UPlanFluo 40×/1.3 NA oil objective (Olympus) was used to visualize labeled viruses. At every time point, two consecutive images were acquired by alternating the standard FITC and TRITC or CY5 excitation/emission filters. Green and orange/red signals were separated by a quad (DAPI/FITC/TRITC/Cy5) dichroic beam-splitter and sequentially acquired using an EM-CCD camera (Photometrics). Unless stated otherwise, virus fusion was monitored by acquiring green and red fluorescence signals at 0.66 Hz (1.5 sec per image) during 35 min. Bulk virus uptake was imaged using a reduced acquisition rate (unless stated otherwise, one frame per 10 sec) for up to 1 hr. The image contrast-based autofocus feature was employed to compensate for a focal drift, which was most noticeable during the first 5–10 min of imaging. In order to provide a reference signal for the autofocus, cell nuclei were labeled with 1 µM Hoechst-33342 and imaged with DAPI filters. When monitoring uptake of Gag-mCherry/EcpH-TM labeled pseudoviruses, cells were pre-stained with MitoTracker Deep Red, and autofocus was implemented using the Cy5 filter.

### Image analyses

All images were deconvoluted using the DeltaVision Softworx package prior to analysis with the Volocity software (Improvision, Waltham, MA). To quantify the global quenching of EcpH signal from multiple viruses entering acidic endosomes, objects (viruses) positive for both Gag-mCherry and EcpH-TM markers were identified in consecutive images and the sum of EcpH signals from all double-labeled objects within the specified size range was calculated. The changes in the mCherry signal were determined by identifying objects positive for this marker, using the parameters applied for double-labeled virions, and the sum of red fluorescence was calculated for every image frame.

The efficacy of EnvA-mediated fusion was evaluated by normalizing the number of virions that released their eGFP content to the total number of double-labeled particles in the image field. The kinetics of single virus entry into acidic compartments (EcpH-TM quenching) and of endosomal fusion (NC-eGFP release) was initially determined based on the visual observation of the onset of EcpH or eGFP fluorescence decay. The time interval from placing the cells into a pre-warmed imaging chamber and the onset of acquisition was subtracted from subsequent quenching or fusion time points. To obtain more quantitative information regarding the fluorescence intensities of the core and membrane markers and to assess the particles' velocities and trajectories, individual viral particles were tracked, as described previously [8]. Due to the poor signal-to-noise ratio and particle crowding at the perinuclear space, only about 30% of double-labeled viruses could be reliably tracked by our software.

The rate of the eGFP fluorescence decay associated with virus fusion was determined by fitting the data with a single exponential function (Sigma Plot, SSI). This analysis yielded the exponential time constant, which was used to calculate the time required to lose half of the eGFP signal (T_50_). In order to minimize the contribution from deterioration of the eGFP signal due to its quenching in acidic compartments, we did not consider the eGFP decay events lasting longer than 5 min. These slow events were predominantly observed in the presence of ASLV fusion inhibitor, R99.

## Supporting Information

Figure S1 Labeling and characterization of ASLV EnvA-pseudotyped viruses. (A) Illustration of the mCherry-TM construct consisting of the mCherry sequence flanked by the triple FLAG-tag and the transmembrane domain of ICAM-1. (B) Immunofluorescence staining of viral particles co-labeled with Gag-eGFP (green) and mCherry-TM (red). Note the inversion of colors of the core and membrane markers compared to Figure 1. This combination of markers was selected in order to ensure the pH-independence of fluorescence of the viral membrane marker. Viruses were immobilized on a poly-lysine-coated chambered coverslip (Lab-Tek™, Rochester, NY) and incubated with anti-Env MC8C5-4 mAb or with isotype control antibodies in a blocking buffer for 1 hr at 4°C. Viruses were then stained with goat anti-mouse IgG conjugated with Cy5™ (colored blue), washed and fixed with 4% paraformaldehyde. (C, D) Analyses of the viral marker colocalization of particles labeled as in panel B and of correlation between the mCherry-TM and Env signals. (E) Immunoprecipitation of double-labeled EnvA-pseudotyped viruses using anti-FLAG M2 and isotype control antibodies. Virions were concentrated by ultracentrifugation and incubated for 2 hr at 4°C with M2-agarose (Sigma) resuspended in HBSS. Viruses remaining in the supernatant after immunoprecipitation were titrated on HEK 293 cells expressing TVA950. (F) Inhibition of EnvA-mediated virus-cell fusion measured by the β-lactamase assay (see Materials and Methods) and plotted as blue/green fluorescence ratio. Fusion of unlabeled viruses and viruses labeled with mCherry-TM was assessed in TVA950-expressing CV-1 cells in the presence or in absence of 50 µg/ml R99 peptide. Data shown are means and SEM from a representative experiment performed in triplicate. G. Western blot analysis of pseudoviruses bearing the subtype A ASLV Env and co-labeled with HIV-1 Gag-mCherry and EcpH-ICAM-1 constructs (designated EnvA-HIV-Gag-mCherry). The bands were probed with either with the 13G4 anti-p24 monoclonal antibody (obtained from the Institute of Human Virology Core Facility) or with a rabbit polyclonal antibody to mCherry (from Clontech). Infectious pNL4-3 HIV-1 preparation was used as control. The Pr55 band on the left panel comes from the wild-type Gag (from the pR8ΔEnv vector) co-expressed with the Gag-mCherry (for details, see Materials and Methods).
(3.32 MB TIF)

Figure S2 TVA800 and TVA950 expression in CV-1 cells. CV-1 cells were transduced with VSV-G pseudotyped retroviral vectors pCMMP-TVA950 or pCMMP-TVA800. Cells expressing either receptor isoform were sorted into high (H) and low (L) fluorescence intensity subpopulations, using a subgroup A ASLV-SU-IgG fusion protein and a FITC-conjugated secondary antibody. The 800L population (not shown) was rather heterogeneous and, therefore, was not used in our experiments. MFI = mean fluorescence intensity.
(2.19 MB TIF)

Figure S3 The lack of increases of membrane permeability of EnvA-pseudotyped viruses upon pretreatment with soluble TVA (sTVA) and low pH. Subtype A Env-pseudotyped viruses were co-labeled with MLV Gag-eGFP and DiD, adhered to a poly-lysine-coated coverslip at 4°C and incubated with 0.5 µg/ml of soluble TVA ectodomain (sTVA) in PBS for 15 min at 37°C or left untreated. Viruses were then exposed to a pH 4.8 MES buffer, and the resulting changes in the eGFP fluorescence of individual particles were monitored using Zeiss LSM 510 Meta confocal microscope. In control experiments, untreated viruses were exposed to a membrane-permeant pH 4.8 acetate buffer. The apparent transient increase in the eGFP fluorescence around 20 sec after addition of MES was due to the focus drift, which was corrected after a few frames.
(0.63 MB TIF)

Figure S4 The kinetics of productive virus uptake and low pH activation in CV-1 cells expressing lower levels of TVA950 (A, 950L) and TVA800 (B, 800L). The rates of receptor-mediated endocytosis (black circles) and EnvA activation in acidic endosomes (red circles) was measured by adding the R99 inhibitory peptide (50 µg/ml) or NH_4_Cl (70 mM), respectively. Virus escape from these inhibitors was assessed by the beta-lactamase-based virus-cell fusion assay (for details, see the legend to Figure 2 and Materials and Methods). The time-course of EcpH-TM quenching (green lines) re-plotted from the Figure 1E is shown as a reference. Data points are means of at least three independent measurements. Error bars are SEM.
(1.18 MB TIF)

Video S1 Imaging of EnvA-pseudotyped virus entry into mildly acidic endosomes. Viruses co-labeled with EcpH-TM (green) and Gag-mCherry (red) were adhered to CV-1 cells expressing TVA950 in the cold, and the temperature was raised to 37°C at time = 0 (elapsed time is shown in the upper right corner). Virus entry into acidic compartment is marked by disappearance of the green signal. A particle in the lower right corner experiences a pH drop without moving considerably from its initial position. Over time, virions move to the perinuclear space that can be identified by somewhat higher autofluorescence compared to the cell periphery. For details, see the legend to Figure 1.
(2.83 MB MOV)

Video S2 Virus recycling and re-internalization. Double-labeled virion (arrowhead) shifts toward the lower left corner of the image and loses its EcpH signal, indicating the virus entry into an acidic compartment. The subsequent recovery of green fluorescence is consistent with virus recycling. The recovery is transient, as the EcpH signal slowly fades again. The time elapsed from shifting CV-1 cells expressing TVA950 and viruses to 37°C is shown on top. For details, see the legend to Figure 3.
(4.53 MB MOV)

Video S3 EnvA-driven virus fusion with an endosome. A virion co-labeled with the membrane marker, DiD (red), and with the diffusible content marker, NC-eGFP (green), travels toward the cell nucleus (stained with Hoechst-33342, blue). The release of the viral content into the cytosol, which is marked by disappearance of the green signal, corresponds to the formation of a fusion pore between the viral and endosomal membranes. The time elapsed after raising the temperature to initiate endocytosis and fusion is shown in the upper right corner. For details, see the legend to Figure 4.
(1.49 MB MOV)
