# Supplementary figures and images for: Imaging Single Retrovirus Entry through Alternative Receptor Isoforms and Intermediates of Virus-Endosome Fusion
Source: PLoS Pathog. 2011 Jan 20;7(1):e1001260. doi: 10.1371/journal.ppat.1001260 (PMC3024281; doi:10.1371/journal.ppat.1001260)

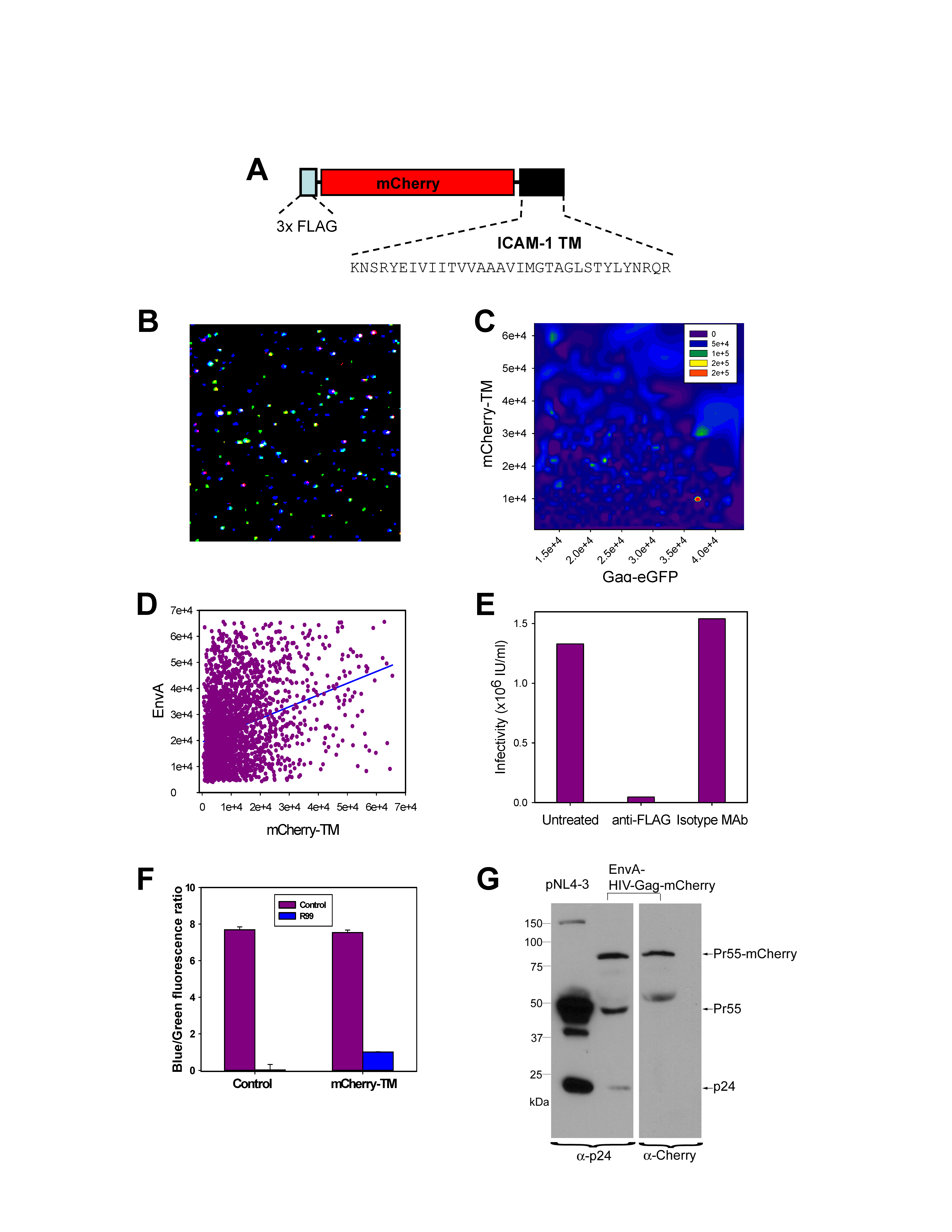

Supplement: Figure S1 — Labeling and characterization of ASLV EnvA-pseudotyped viruses. (A) Illustration of the mCherry-TM construct consisting of the mCherry sequence flanked by the triple FLAG-tag and the transmembrane domain of ICAM-1. (B) Immunofluorescence staining of viral particles co-labeled with Gag-eGFP (green) and mCherry-TM (red). Note the inversion of colors of the core and membrane markers compared to Figure 1. This combination of markers was selected in order to ensure the pH-independence of fluorescence of the viral membrane marker. Viruses were immobilized on a poly-lysine-coated chambered coverslip (Lab-Tek™, Rochester, NY) and incubated with anti-Env MC8C5-4 mAb or with isotype control antibodies in a blocking buffer for 1 hr at 4°C. Viruses were then stained with goat anti-mouse IgG conjugated with Cy5™ (colored blue), washed and fixed with 4% paraformaldehyde. (C, D) Analyses of the viral marker colocalization of particles labeled as in panel B and of correlation between the mCherry-TM and Env signals. (E) Immunoprecipitation of double-labeled EnvA-pseudotyped viruses using anti-FLAG M2 and isotype control antibodies. Virions were concentrated by ultracentrifugation and incubated for 2 hr at 4°C with M2-agarose (Sigma) resuspended in HBSS. Viruses remaining in the supernatant after immunoprecipitation were titrated on HEK 293 cells expressing TVA950. (F) Inhibition of EnvA-mediated virus-cell fusion measured by the β-lactamase assay (see Materials and Methods) and plotted as blue/green fluorescence ratio. Fusion of unlabeled viruses and viruses labeled with mCherry-TM was assessed in TVA950-expressing CV-1 cells in the presence or in absence of 50 µg/ml R99 peptide. Data shown are means and SEM from a representative experiment performed in triplicate. G. Western blot analysis of pseudoviruses bearing the subtype A ASLV Env and co-labeled with HIV-1 Gag-mCherry and EcpH-ICAM-1 constructs (designated EnvA-HIV-Gag-mCherry). The bands were probed with either with the 13G4 [file ppat.1001260.s001.tif]

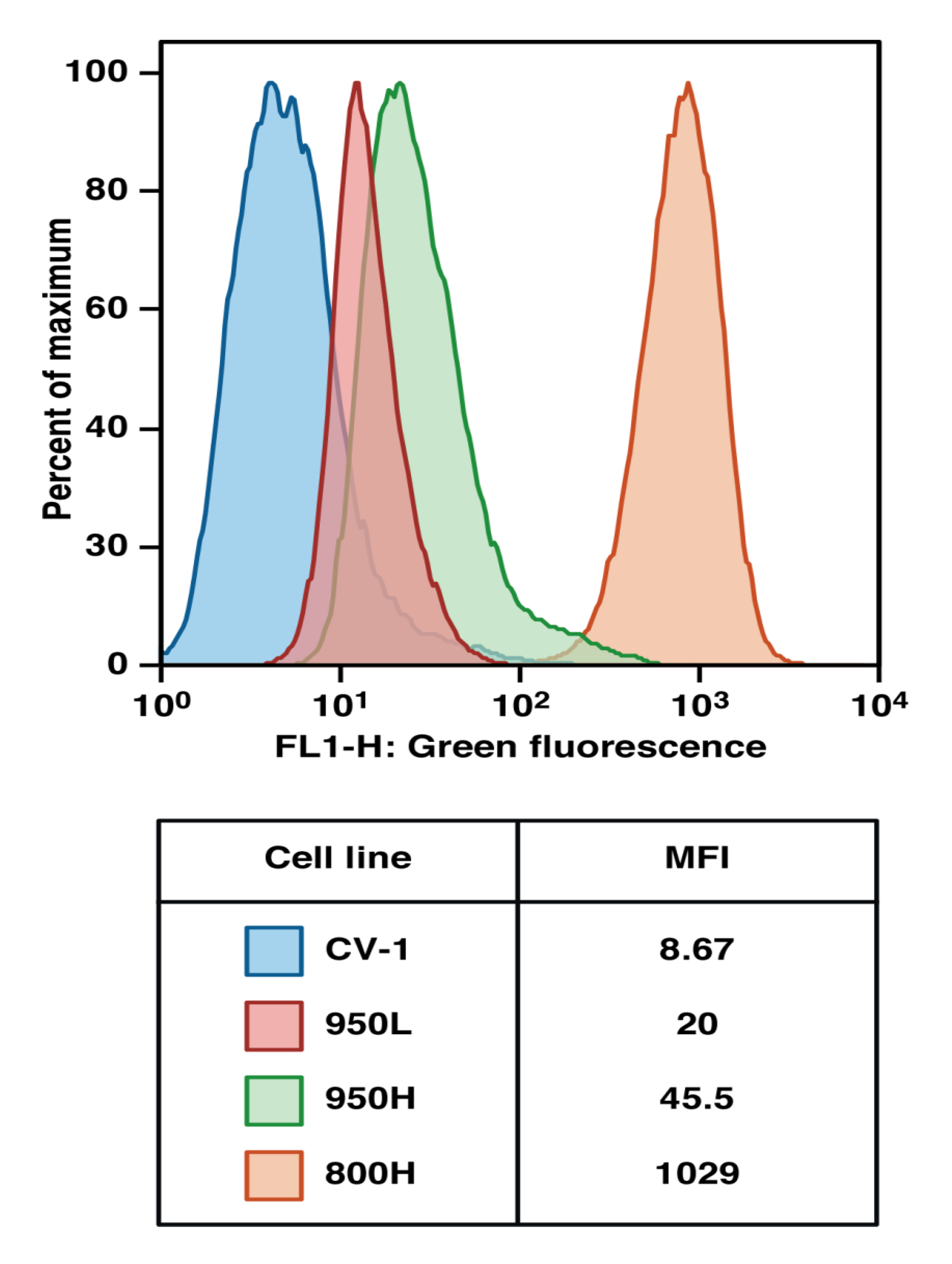

Supplement: Figure S2 — TVA800 and TVA950 expression in CV-1 cells. CV-1 cells were transduced with VSV-G pseudotyped retroviral vectors pCMMP-TVA950 or pCMMP-TVA800. Cells expressing either receptor isoform were sorted into high (H) and low (L) fluorescence intensity subpopulations, using a subgroup A ASLV-SU-IgG fusion protein and a FITC-conjugated secondary antibody. The 800L population (not shown) was rather heterogeneous and, therefore, was not used in our experiments. MFI = mean fluorescence intensity. (2.19 MB TIF) [file ppat.1001260.s002.tif]

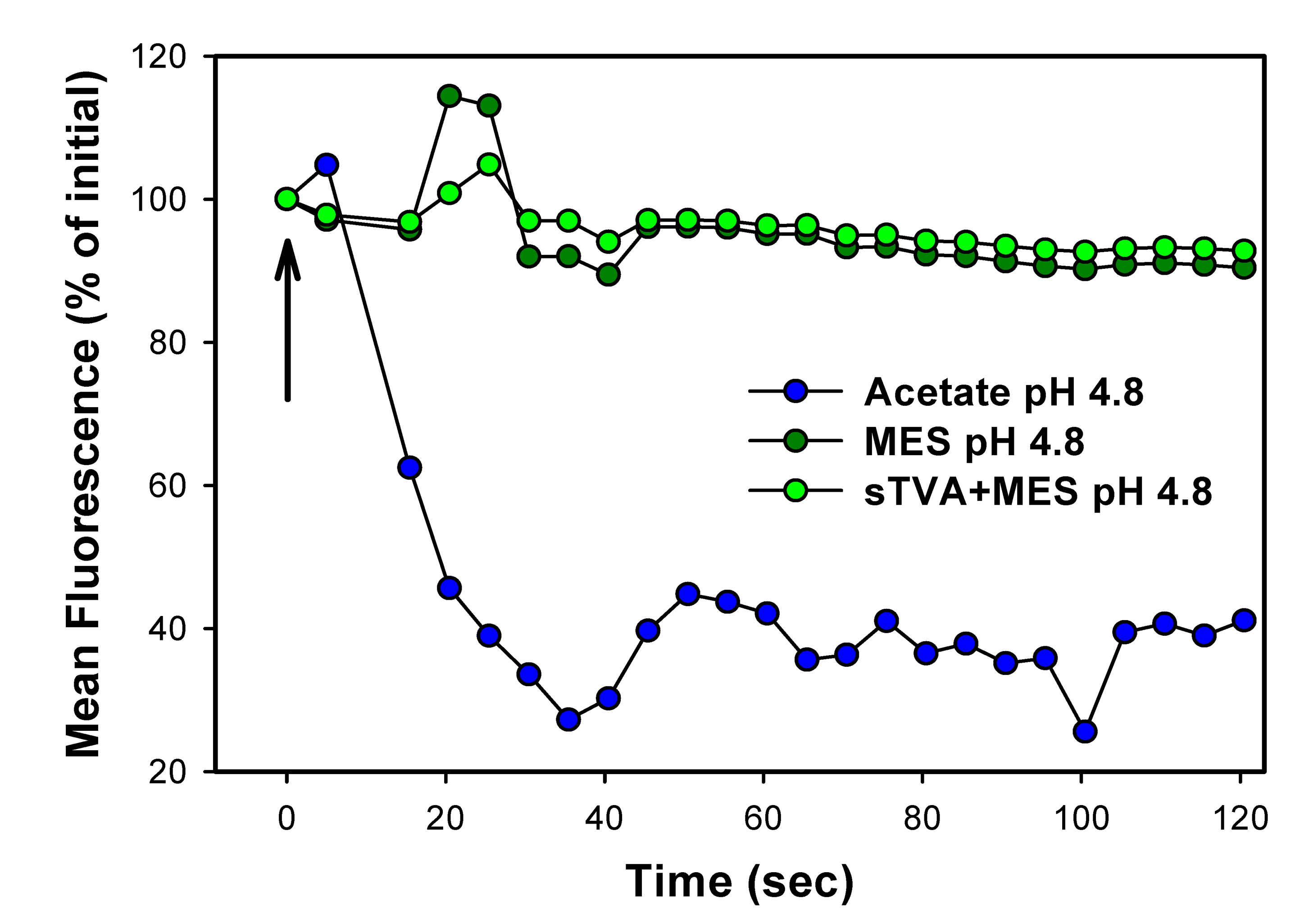

Supplement: Figure S3 — The lack of increases of membrane permeability of EnvA-pseudotyped viruses upon pretreatment with soluble TVA (sTVA) and low pH. Subtype A Env-pseudotyped viruses were co-labeled with MLV Gag-eGFP and DiD, adhered to a poly-lysine-coated coverslip at 4°C and incubated with 0.5 µg/ml of soluble TVA ectodomain (sTVA) in PBS for 15 min at 37°C or left untreated. Viruses were then exposed to a pH 4.8 MES buffer, and the resulting changes in the eGFP fluorescence of individual particles were monitored using Zeiss LSM 510 Meta confocal microscope. In control experiments, untreated viruses were exposed to a membrane-permeant pH 4.8 acetate buffer. The apparent transient increase in the eGFP fluorescence around 20 sec after addition of MES was due to the focus drift, which was corrected after a few frames. (0.63 MB TIF) [file ppat.1001260.s003.tif]

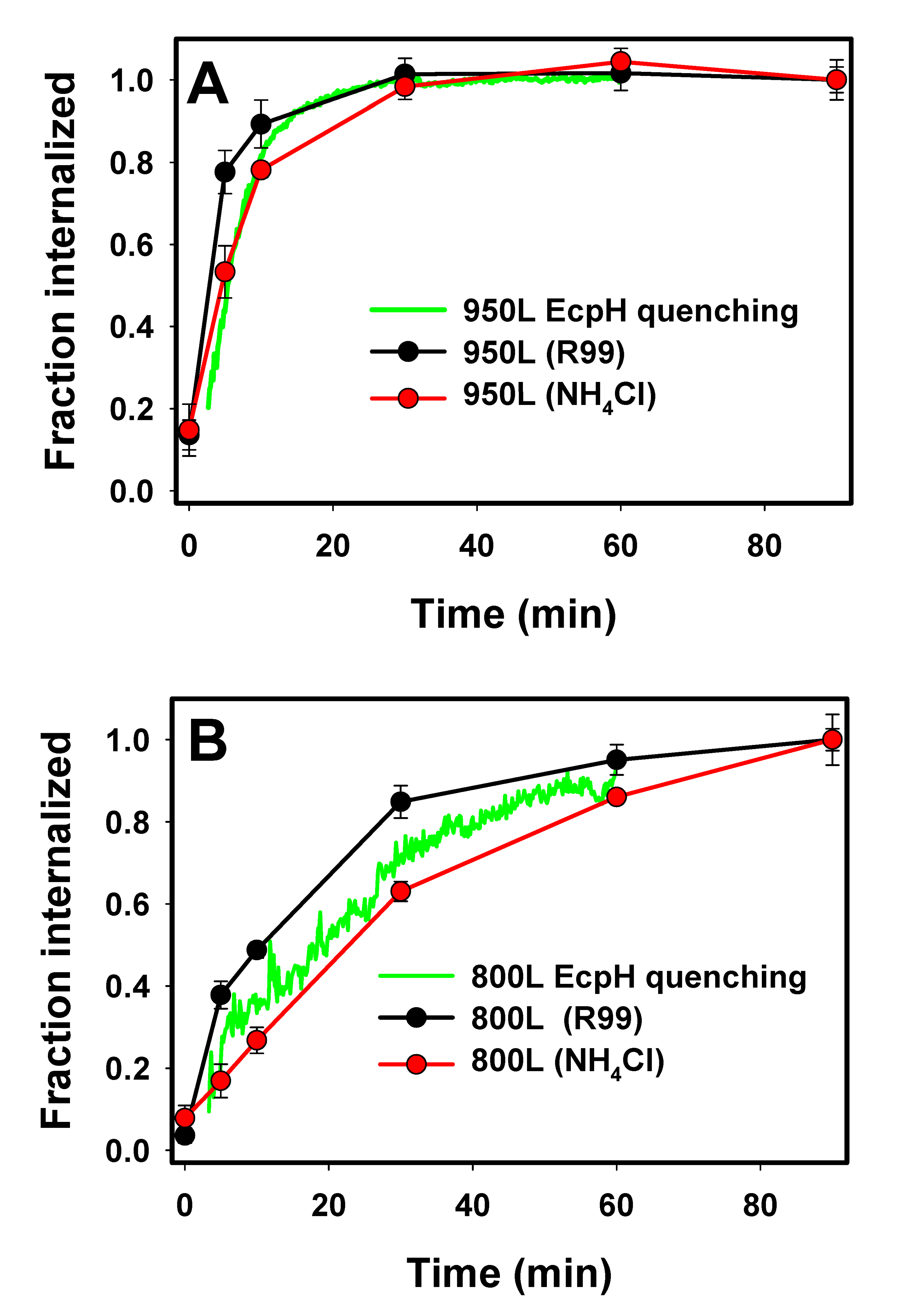

Supplement: Figure S4 — The kinetics of productive virus uptake and low pH activation in CV-1 cells expressing lower levels of TVA950 (A, 950L) and TVA800 (B, 800L). The rates of receptor-mediated endocytosis (black circles) and EnvA activation in acidic endosomes (red circles) was measured by adding the R99 inhibitory peptide (50 µg/ml) or NH4Cl (70 mM), respectively. Virus escape from these inhibitors was assessed by the beta-lactamase-based virus-cell fusion assay (for details, see the legend to Figure 2 and Materials and Methods). The time-course of EcpH-TM quenching (green lines) re-plotted from the Figure 1E is shown as a reference. Data points are means of at least three independent measurements. Error bars are SEM. (1.18 MB TIF) [file ppat.1001260.s004.tif]
